# Case report: Extraskeletal mesenchymal chondrosarcoma with a rare metastasis to the pancreas

**DOI:** 10.3389/fonc.2024.1324732

**Published:** 2024-10-09

**Authors:** Xiuliang Zhu, Lu Cheng, Fei Dong, Jinsong Cai, Wei Qian, Qiao-Ling Ding

**Affiliations:** ^1^ Department of Radiology, the Second Affiliated Hospital, Zhejiang University School of Medicine, Hangzhou, China; ^2^ Department of Pathology, the Second Affiliated Hospital, Zhejiang University School of Medicine, Hangzhou, China; ^3^ Department of Radiology, Hangzhou Xixi Hospital Affiliated to Zhejiang Chinese Medical University, Hangzhou, China

**Keywords:** soft tissue neoplasms, mesenchymal chondrosarcoma, metastasis, contrast-enhanced computed tomography, magnetic resonance imaging, case report

## Abstract

**Background:**

Extraskeletal mesenchymal chondrosarcoma (ESMC), an uncommon and highly aggressive form of chondrosarcoma, is characterized by its mesenchymal origin and absence of skeletal involvement. Only a few cases of primary ESMC with metastasis to the pancreas have been reported so far. In this study, we present a case of ESMC in the left thigh with a solitary pancreatic metastasis in a 45-year-old woman. Additionally, we provide a thorough overview of ESMC, encompassing its entire clinical progression and radiographic observations. Furthermore, we reviewed all thirteen cases of pancreatic metastasis, including this present case, analyzing patient attributes, clinical management, and prognosis.

**Case presentation:**

A 45-year-old woman has had a painless mass in her left thigh for one year. X-ray, computed tomography (CT), and magnetic resonance imaging of the left thigh were performed. Positron emission tomography-CT imaging showed a high accumulation in the left thigh tumor and the pancreatic neck lesion. A diagnosis of extraskeletal chondrosarcoma with pancreatic metastasis was determined based on the radiological examinations. A final diagnosis of ESMC was confirmed by histopathological and immunohistochemical examinations after surgical resection. The patient presented metastasis in the lung, right groin, and tail of the pancreas successively, and mostly received complete surgical excision during a 39-month follow-up with postoperative chemotherapy.

**Conclusion:**

We present a highly uncommon case of ESMC spreading to the pancreas and highlight the importance of recognizing the distinctive imaging features of ESMC for diagnosis and prognosis assessment.

## Introduction

Chondrosarcomas, which generate a cartilaginous matrix, are a diverse set of cancerous growths, with extraskeletal origins being much rarer than their intraosseous counterparts (1). The mesenchymal subtype, comprising 2% to 9% of initial chondrosarcomas, is highly uncommon (2, 3). Unlike typical chondrosarcoma, mesenchymal chondrosarcoma (MC) primarily affects young adults in their twenties and thirties (4), with no significant gender preference (5). Extraskeletal mesenchymal chondrosarcoma (ESMC) can originate from either the bone or soft tissues, with reported proportions ranging from 10% to 60% (1, 8, 9). Most of them are located in the lower limbs (especially the thigh), meninges, soft tissues of the chest and abdominal wall, mediastinum, and the orbit (5). Owing to its heightened aggressiveness, MC has been linked to a worse prognosis and a strong tendency to metastasize, exhibiting 5-year and 10-year survival rates of only 51% and 43%, respectively. There was no distinction observed between extraskeletal and skeletal locations. Nevertheless, there was significant variation depending on the anatomical sites, as evidenced by the 5-year survival rates of 74% for cranial tumors, 50% for appendicular tumors, and 37% for axial tumors (3).

The metastasis of ESMC to the pancreas is an exceptionally uncommon occurrence. We report a case of left thigh ESMC with pancreatic metastasis and review all the published literature on twelve cases of pancreatic metastasis from ESMC (1, 2, 8–17). This information is primarily found in case reports or case series. Our aim is to gather the data published so far to highlight the number of reported pancreatic metastases, imaging characteristics, status of genetic diagnosis, treatment, and prognosis.

## Case presentation

### History and examination

Our institution received a 45-year-old female patient who was referred due to a painless lump in her left thigh that had been present for 1 year. The physical examination showed a mass of approximately 10 cm×8 cm in size located at the back of the left thigh. She had unremarkable surgical and family histories.

### Radiological examinations

**Figure 1:**
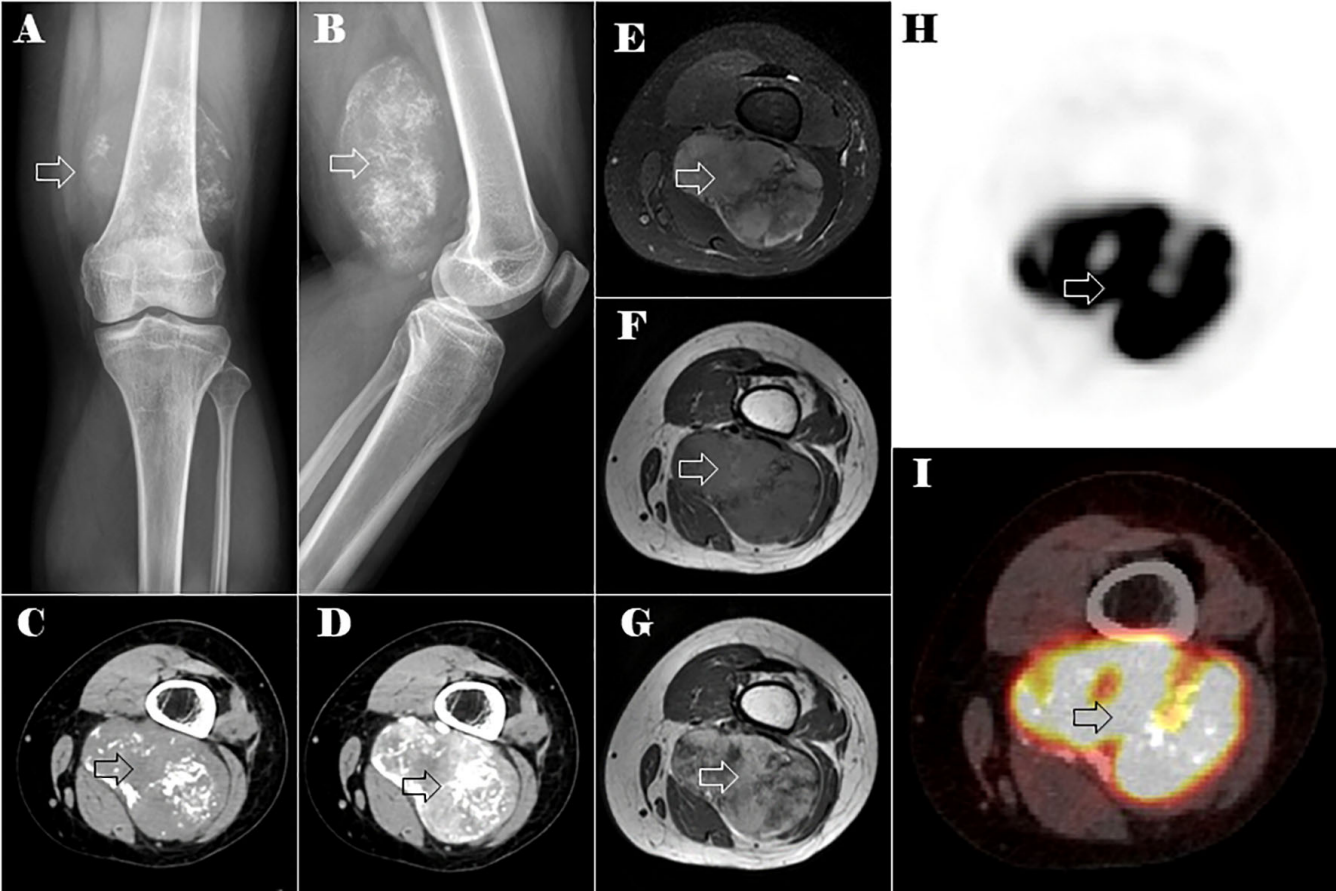
Plain X-ray, CT, MRI, and PET-CT images of the lesion in the left distal thigh (the lesion is indicated by the hollow arrow). Anteroposterior **(A)** and lateral **(B)** plain x-ray showed an oval soft tissue mass with spotty calcifications. Axial CT of soft tissue window images in the unenhanced **(C)** and enhanced **(D)** phases. Axial MRI images of the tumor mass on non-contrast T1-weighted **(E)**, T2-weighted fat-suppression **(F)**, and T1 enhanced **(G)** images. Axial PET-CT images of the lesions in the left distal thigh **(H, I)**.

**Figures 1A, B** shows radiographs of the left thigh, revealing a oval soft tissue lump with scattered calcifications. A computed tomography (CT) scan of the left thigh revealed a regularly lobulated well-circumscribed mass measuring 9 cm×6 cm in the muscular portion of the left posterior thigh. The tumor exhibited slightly lower and heterogeneous density with internal irregular calcifications, displaying a chondroid pattern resembling rings and arcs. An enhanced CT scan demonstrated septal and peripheral enhancement around the calcified area, accompanied by more heterogeneous or diffuse enhancement regions in the non-calcific area of the lesion (**Figures 1C, D**). Moreover, magnetic resonance imaging (MRI) of the left thigh revealed a tumor mass with heterogeneous hypointensity on the non-contrast T1-weighted image and heterogeneous hyperintensity on the T2-weighted fat-suppression image, both showing varying levels of irregular low signal. The T1 enhanced image demonstrated enhancement features comparable with those observed on CT (**Figures 1E–G**). Based on these imaging findings, a provisional diagnosis of extraskeletal chondrosarcoma was considered. Differential diagnosis included Ewing’s sarcoma, osteosarcoma, and synovial sarcoma.

**Figure 2:**
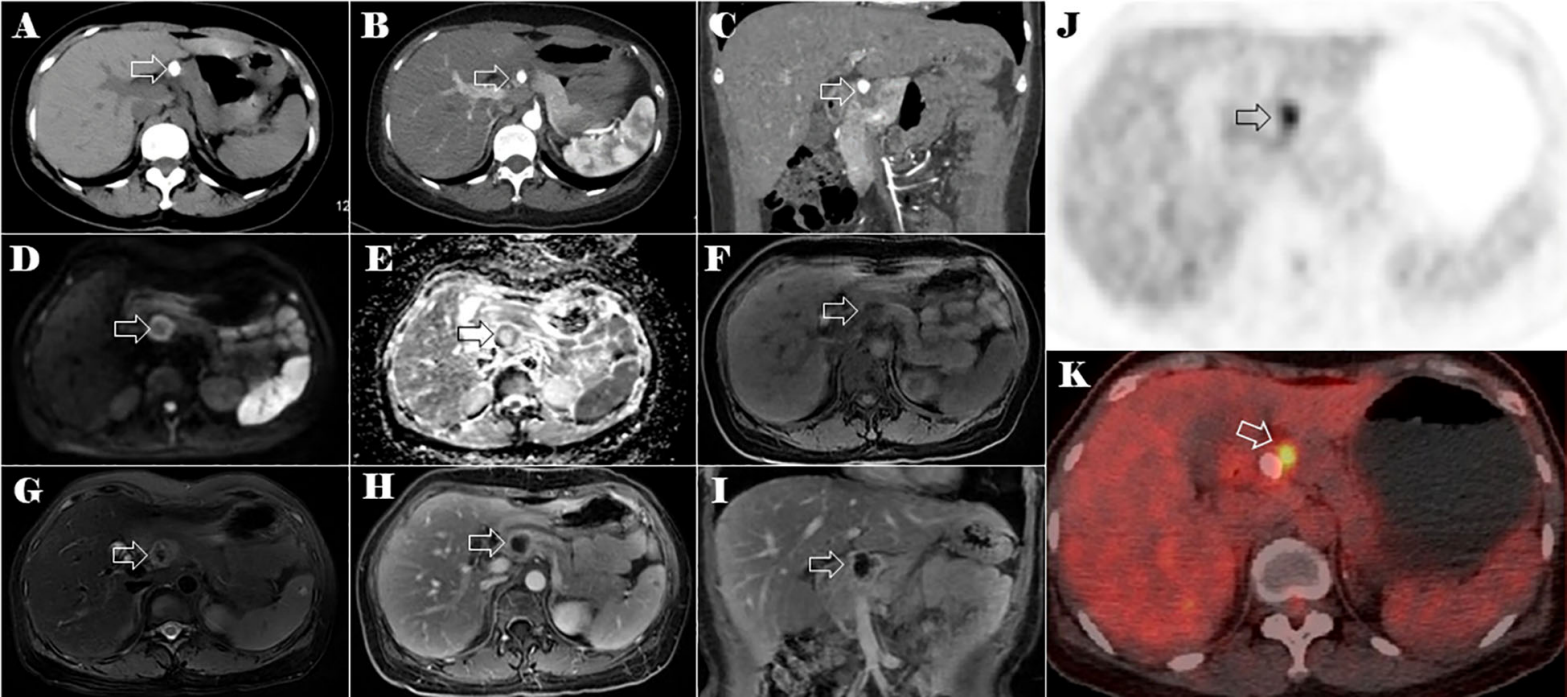
CT, MRI, and PET-CT images of the lesion in the neck of the pancreas (the lesion is indicated by the hollow arrow). Plain CT axial image **(A)**, arterial phase of contrast-enhanced CT axial **(B)** and coronal **(C)** images of the abdomen. The axial multi-sequence MRI images of the pancreas on DWI **(D)**, a ADC* image **(E)**, a T1-weighted image **(F)**, a T2-weighted fat-suppression image **(G)**, and a T1 enhanced image **(H)**. Coronal T1 enhanced image **(I)** of the lesion in the neck of the pancreas. Axial PET-CT images of the lesions in the neck of the pancreas **(J, K)**.

The abdominal CT scan identified a calcified mass in the neck of the pancreas, measuring approximately 2.4 cm in diameter. The mass appeared well-defined, with low attenuation and heterogeneous enhancement. There was no evidence of distal pancreatic duct dilatation or surrounding tissue invasion (**Figures 2A–C**). The lesion exhibited peripheral ring-like high signal intensity on the diffusion-weighted imaging (DWI) and low signal intensity on the apparent diffusion coefficient (ADC) map. On T1-weighted images, the mass appeared hypointense with punctate areas of hyperintensity, whereas on the T2-weighted fat-suppression image, it showed slight hyperintensity. The tumor exhibited irregular enhancement, with the most pronounced enhancement at its periphery (**Figures 2D–I**). Given the patient’s history of a tumor in the left thigh, metastasis to the pancreatic neck was strongly suspected.

Positron emission tomography-CT (PET-CT) imaging showed elevated maximum standardized uptake values (SUVs) of fluorodeoxyglucose in the tumor of the left thigh (maximum SUV, 39.58) (**Figures 1H, I**) and the lesion in the neck of the pancreas (maximum SUV, 5.92) (**Figures 2J, K**).

### Results from the surgical exploration and pathological analysis

**Figure 3:**
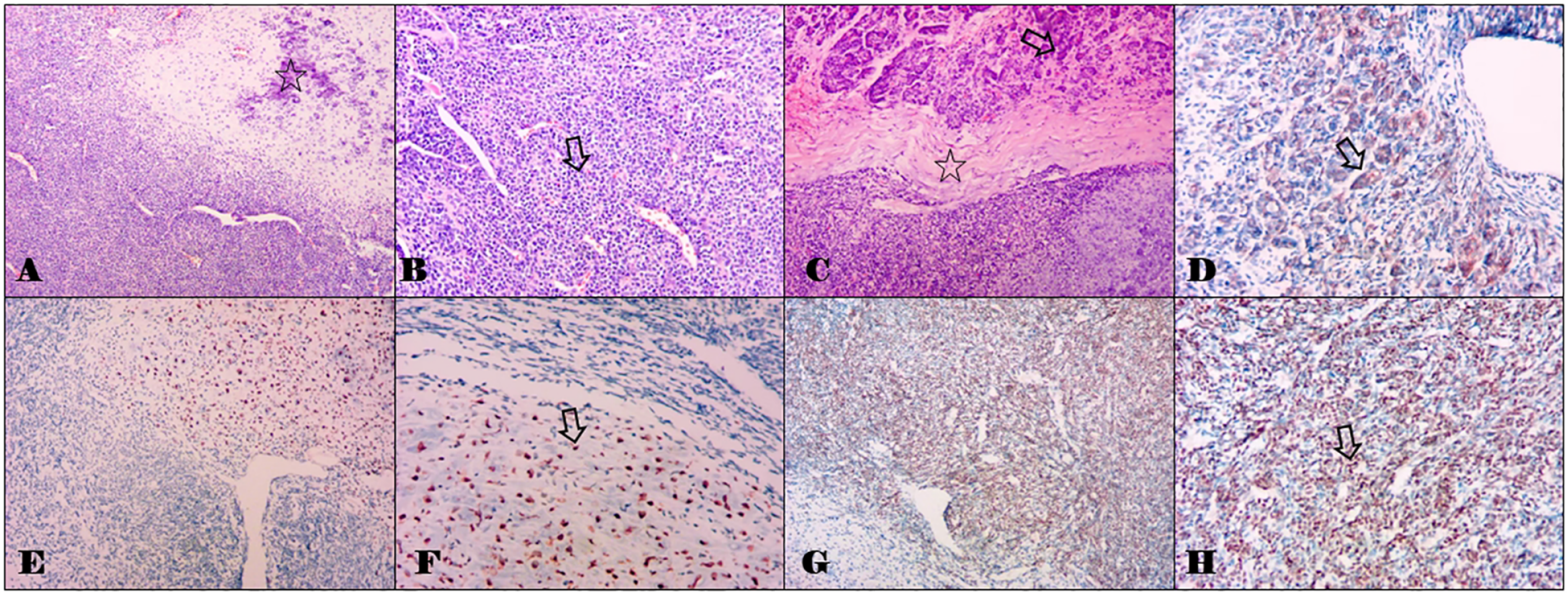
Histomorphological staining results of the tumors in the thigh and the pancreas with hemotoxylin and eosin, and partial immunohistochemical staining results of the pancreatic metastasis. **(A)** Combination of cartilaginous islands and undifferentiated small cells with an abrupt transition between them. The hollow star indicates the cartilaginous islands (0riginal magnification, ×100). **(B)** The component of the primitive undifferentiated mesenchymal cell shows a small round to oval cellular appearance with scant cytoplasm bearing a considerable resemblance to myopericytoma with numerous vascular clefts. The hollow arrow indicates small round cells (original magnification, ×200). **(C)** The metastatic tumor in the pancreas morphologically resembles that in the thigh **(A, B)**. The hollow arrow indicates the normal pancreas tissues, and the hollow star indicates the chondroid matrix. (original magnification, ×100). **(D)** Immunohistochemical staining with CD99 in a cartilaginous island. The hollow arrow indicates positive staining (original magnification, ×200). **(E, F)** Immunohistochemical staining with S-100. The hollow arrow indicates positive staining (original magnification, ×100 and ×200). **(G, H)** Immunohistochemical staining with NKX 3.1. The hollow arrow indicates positive staining (original magnification, ×100 and ×200).

The tumor in her thigh was completely removed, and histopathologic examination revealed a malignant mesenchymal neoplasm composed of two distinct cell types. One type consisted of undifferentiated primitive mesenchymal cells, characterized by a short spindle or oval shape and minimal cytoplasm. The other type presented as well-developed islands of hyaline cartilage (**Figure 3**). Based on the microscopic appearance, a histopathologic diagnosis of primary ESMC was made. One month later, she underwent pancreatic surgery, and intraoperative examination confirmed the tumor’s location in the neck of the pancreas, without infiltration into surrounding tissues or peritoneal dissemination. The pancreatic tumor was successfully resected. Pathological analysis revealed tumor tissue with osteochondroid differentiation. Immunohistochemistry results showed NKX3.1 positivity and focal CD99 positivity (weak +), whereas S-100, NSE, P53, Desmin, SMA, STAT6, and SOX10 were negative. Ki67 was positive in 20% of cells, and CD34 showed positivity in the vascular component. Considering the clinical history, the diagnosis of metastasis of the ESMC to the pancreatic neck was made.

### Post-operative course and follow-up

**Figure 4:**
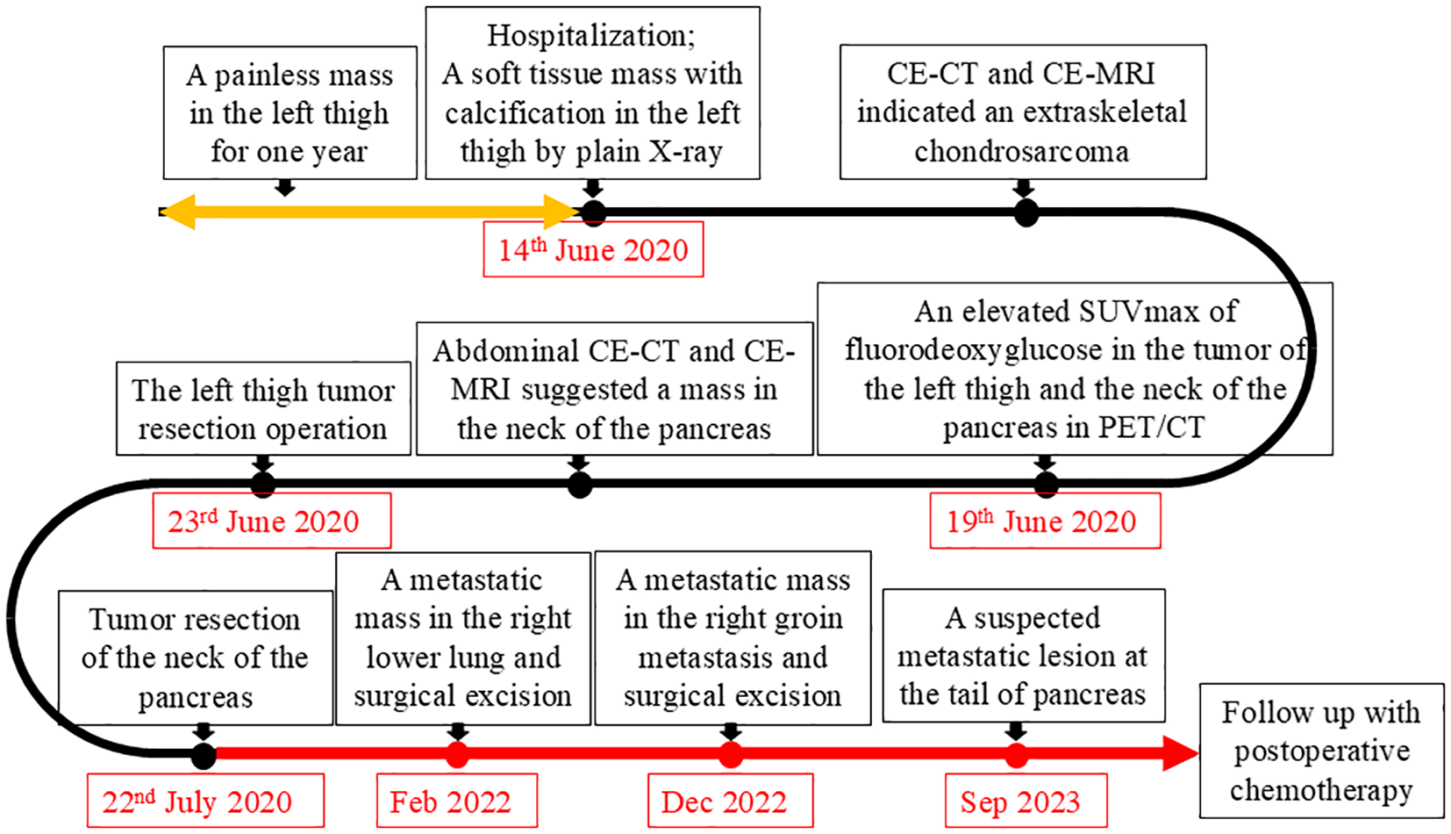
The timeline of diagnosis and treatment.

Post-surgery, the patient received VAC/IE chemotherapy, consisting of vincristine, adriamycin, cyclophosphamide, ifosfamide, and etoposide in alternating cycles. Despite this treatment, she developed metastases in the right lower lung and right groin, which were surgically excised 20 months and 30 months after the initial tumor resection, respectively. At the latest follow-up on 20 September 2023, a suspected metastatic lesion was detected in the pancreatic tail. Currently, the patient is alive. **Figure 4** illustrates the timeline of diagnosis and treatment.

## Discussion

Chondrosarcoma, a malignancy with cartilaginous differentiation, is an uncommon tumor that primarily arises in skeletal or cartilaginous structures. Additionally, it can manifest in locations outside of the skeletal system where cartilage is typically absent. Extraskeletal chondrosarcoma can be categorized into myxoid, mesenchymal, and well-differentiated histological subtypes. Typically, the myxoid subtype is prevalent, whereas the mesenchymal subtype is exceedingly uncommon (18).

Traditionally, the identification of MC relied on its distinctive histological characteristics. The classic histologic appearance of MCs shows a biphasic tumor made up of sheets and nests of primitive-appearing small round blue cells surrounding irregular islands of hyaline cartilage. Although MCs demonstrate immunohistochemical staining with CD99 and S100, these markers are relatively nonspecific and can show positive staining in several tumors that may be included in the differential diagnosis (19, 20). The primary lesion and pancreatic metastasis in our reported case are consistent with previously reported findings. Nevertheless, this can be challenging when the biphasic morphology is not apparent, such as when one component of the tumor is more dominant or due to sampling bias or limited tissue availability during biopsy. In 2012, the *HEY1*:*NCOA2* fusion transcript was discovered as a potentially useful diagnostic tool for MC. This novel fusion can help differentiate MC from other (chondro-)sarcomas in difficult or uncertain cases (21). Cohen et al. identified two cases with pancreatic metastasis of eight MC patients of bone (25%), and both pancreatic tumors harbored the *HEY1*:*NCOA2* gene fusion (20). Ghafoor et al. revealed that out of the twelve cases, three (25%) had metastasis to the pancreas, and among the six patients, four were found to be positive for the *HEY1*:*NCOA2* rearrangement (7). We reviewed all 13 reported cases of ESMC with pancreatic metastasis (including our case), two of which were primary pancreatic tumors. Of the six cases reported after the discovery of the *HEY1*:*NCOA2* gene fusion, only one tested positive for the *HEY1*:*NCOA2* fusion gene (8, 28) (**Supplementary Table S1**). Therefore, additional research is needed to validate the presence of the *HEY1*:*NCOA2* gene fusion in all pancreatic ESMCs.

The use of imaging is crucial in the management and precise diagnosis of MC. In our case, the main ESMC in the left thigh showed an internal irregular pattern of calcifications resembling rings and arcs of chondroid type, whereas the pancreatic metastasis exhibited central calcifications surrounded by a non-calcified tumor periphery. A study by Ghafoor et al., which included 23 MC patients (13 skeletal and 10 extraskeletal), analyzed the features of calcification in MC. The study suggested a unique biphasic structure, in which a mass is divided into a calcified and non-calcified component by a well-defined transition zone (7). In their cohort, this characteristic form was found in 30% of cases. In the study by Hashimoto et al., it was observed in 40% of patients (22), and it can also be observed in previous studies (18, 20, 22–24). The calcification characteristics of the primary and metastatic masses in our case exhibit the distinct biphasic morphology. Therefore, the dual-phase structure could serve as a valuable diagnostic indication, and it is important to note for guiding biopsy preparation and subsequent management strategies.

Non-specific MR signal characteristics of MC have been summarized in several studies. In our case, both the primary ESMC in the left thigh and the pancreatic metastasis exhibited a low signal on T1-weighted images and a heterogeneously hyperintense signal on T2-weighted images. The degree of heterogeneity varies based on the quantity and distribution of calcifications, which caused low T2 signals. Interestingly, the calcifications in a rings-and-arcs pattern within the main tumor did not correspond directly to a distinctive chondroid appearance on T2-weighted MRI. These MR signal characteristics align with previously published studies (7, 22, 25).

The enhancement patterns of MC were diverse, with chondroid-type enhancement being the most common, characterized by septal and peripheral enhancement (7). Our case, along with most patients in previous studies, demonstrated this pattern (7, 22, 25). Notably, this enhancement pattern was observed even if it appeared only in certain regions of the tumor. Typically, there was a mix of non-chondroid and more varied or widespread enhancement areas. Additionally, the mass displayed variations in enhancement due to its biphasic morphology: the non-calcified sections exhibited more uniform and noticeable enhancement, whereas the calcified areas corresponded to a lack of enhancement.

Compared with typical chondrosarcomas, MC tends to occur in younger individuals and frequently metastasizes. Research by Ghafoor et al. found that the lungs were the most likely site for metastases, occurring in 75% of cases. Uncommon sites, including the pancreas (25%) and kidneys (17%), were observed in 42% of cases. Metastatic disease developed in approximately 52% of cases, resulting in a 30% mortality rate. There was no significant difference in the metastasis occurrence and mortality rate between ESMC (50% and 33.3%, respectively) and skeletal MC (53.8% and 31%, respectively) (7). These findings align closely with previously published data (6, 20, 22, 26). We conducted a retrospective review of all the literature on ESMC and its metastases (excluding pancreatic metastases) (**Supplementary Table S1**). Our findings show a metastasis rate of approximately 46.3% (148 of 320 cases) for MC and 52.7% (19 of 36 cases) for ESMC, consistent with previous reports (**Supplementary Tables S1**, **S2**) (29–36). A study of 5,110 individuals diagnosed with chondrosarcoma in the SEER database revealed that MC represented 4% of cases, with 5- and 10-year survival rates of 51% and 43%, respectively. There was no significant difference in overall survival between extraskeletal and skeletal MC (3). However, survival rates varied by anatomical site: cranial tumors had a 5-year overall survival rate of 74%, appendicular tumors 50%, and axial tumors 37%. Poor survival outcomes were primarily associated with the presence of metastatic disease and an enlarged tumor size (3). In general, the prognosis for MC is less favorable than for classical chondrosarcoma, but is not as grim as some previous studies have suggested (26, 27).

Owing to the uncommon and unpredictable characteristics of MC, its management has not been thoroughly researched. However, the existing consensus on management highlights the significance of controlling the disease both locally and systemically (26). Wide surgical resection is the primary approach for local treatment, backed by evidence indicating increased survival rates in patients who undergo surgery (27). When the disease is inoperable, the simultaneous application of radiotherapy and chemotherapy might be beneficial, even though there is still a lack of compelling evidence (6, 7, 27).

To our knowledge, only 12 such cases have been documented in the medical literature (**Supplementary Table S1**). Therefore, our case should be considered the 13th reported case of pancreatic ESMC. The age range at onset is 24–45 years, with no significant gender difference, comprising six males and seven females. The largest sizes of pancreatic metastases ranges from 2 cm to 18 cm, with 10 out of 13 tumors primarily located in the body and tail of the pancreas. Calcification within the pancreatic tumor was found in seven of the reported cases. The average latency period for pancreatic metastasis after the primary tumor diagnosis is approximately 5.5 years. There are currently no specific treatment guidelines for this type of tumor. In the reported cases, most treatment strategies involved surgery and further systemic chemotherapy. Additionally, radiation therapy has been recorded as a treatment strategy (2). In our case, the patient underwent resection of the primary tumor and the pancreatic metastasis followed by chemotherapy, yet still developed multiple recurrences. This underscores the importance of long-term follow-up after successful treatment.

## Conclusion

In summary, ESMC with metastasis to the pancreas is rare. The radiological features are non-specific but the biphasic shape and chondroid-like enhancement can serve as useful diagnostic indicators, particularly for tumors that develop in the pancreas. Additional research is needed to validate the presence of the *HEY1*:*NCOA2* gene fusion in all pancreatic ESMCs. Owing to its rarity, MC management lacks extensive research but wide surgical resection is the primary treatment, and combined radiotherapy and chemotherapy may offer benefits. The prognosis for ESMC is more unfavorable than for classical chondrosarcoma, although it does not appear to be as bleak as indicated by certain previous studies.

## Data Availability

The original contributions presented in the study are included in the article/**Supplementary Material**. Further inquiries can be directed to the corresponding author.

## Supplementary material

Supplementary Table 1 Literature review of the published cases of primary/metastatic ESMC of the pancreas. (highlighted) added in this report. ***** Outcome: months or years of survival after surgery. ****** Years of survival after the diagnosis of MC. △ *HEY1*:*NCOA2* gene fusion was first identified in MC in 2012 (21). **★ C**ases of the primary ESMC of the pancreas. CT, chemotherapy; RT, radiotherapy; NR, not recorded; NA, not applicable.

Supplementary Table 2 Literature review of the number of published skeletal and extraskeletal MC and the duration of follow-up for ESMC metastasis (Pancreatic metastasis not included). △ The numbers in parentheses on the right indicate the total number of MC cases followed up, while the numbers on the left represent the cases in which metastasis occurred, including both skeletal and extraskeletal origins. ★ The number of cases in which metastasis occurred among all cases of MC, including both skeletal and extraskeletal origins. NR, not recorded. NA, not applicable.

## References

[1] Guo J Gu Y Guo L Tong Z Wu X Zhang J. A case of mesenchymal chondrosarcoma arising from the femoral vein with 8 years of follow-up. Ann Vasc Surg. (2015) 29:1451–55. doi: 10.1016/j.avsg.2015.04.086 26133997

[2] Naumann S Krallman PA Unni KK Fidler ME Neff JR Bridge JA. Translocation der (13;21) (q10;q10) in skeletal and extraskeletal mesenchymal chondrosarcoma. Mod Pathol. (2002) 15:572–76. doi: 10.1038/modpathol.3880565 12011263

[3] Schneiderman BA Kliethermes SA Nystrom LM. Survival in mesenchymal chondrosarcoma varies based on age and tumor location: a survival analysis of the SEER database. Clin Orthop Relat Res. (2017) 475:799–805. doi: 10.1007/s11999-016-4779-2 26975384 PMC5289165

[4] Gelderblom H Hogendoorn PC Dijkstra SD van Rijswijk CS Krol AD Taminiau AH. The clinical approach towards chondrosarcoma. Oncologist. (2008) 13:320–29. doi: 10.1634/theoncologist.2007-0237 18378543

[5] Nakashima Y Unni KK Shives TC Swee RG Dahlin DC. Mesenchymal chondrosarcoma of bone and soft tissue. A review of 111 cases. Cancer. (1986) 57:2444–53. doi: 10.1002/(ISSN)1097-0142 3697943

[6] Shakked RJ Geller DS Gorlick R Dorfman HD. Mesenchymal chondrosarcoma: clinicopathologic study of 20 cases. Arch Pathol Lab Med. (2012) 136:61–75. doi: 10.5858/arpa.2010-0362-OA 22208489

[7] Ghafoor S Hameed MR Tap WD Hwang S. Mesenchymal chondrosarcoma: imaging features and clinical findings. Skeletal Radiol. (2021) 50:333–41. doi: 10.1007/s00256-020-03558-x PMC8491146 32734374

[8] Hayashida S Ikenaga N Nakata K Nakamura S Abe T Ideno N. Repeated robotic pancreatectomy for recurrent pancreatic metastasis of mesenchymal chondrosarcoma: a case report. Asian J Endosc Surg. (2023) 16:795–99. doi: 10.1111/ases.13240 37574440

[9] Chen JJ Chou CW. A rare case report of mesenchymal chondrosarcoma with pancreatic metastasis. Medicina (Kaunas). (2022) 58:639. doi: 10.3390/medicina58050639 PMC9144319 35630056

[10] Smith AL Odronic SI Springer BS Reynolds JP. Solid tumor metastases to the pancreas diagnosed by FNA: a single-institution experience and review of the literature. Cancer Cytopathol. (2015) 123:347–55. doi: 10.1002/cncy.21541 25828394

[11] Tsukamoto S Honoki K Kido A Fujii H Enomoto Y Ohbayashi C. Chemotherapy improved prognosis of mesenchymal chondrosarcoma with rare metastasis to the pancreas. Case Rep Oncol Med. (2014) 2014:249757. doi: 10.1155/2014/249757 24716041 PMC3970347

[12] Bu X Dai X. Primary mesenchymal chondrosarcoma of the pancreas. Ann R Coll Surg Engl. (2010) 92:W10–12. doi: 10.1308/147870810X12659688851672 PMC5696814 20412660

[13] Oh BG Han YH Lee BH Kim SY Hwang YJ Seo JW. Primary extraskeletal mesenchymal chondrosarcoma arising from the pancreas. Korean J Radiol. (2007) 8:541–44. doi: 10.3348/kjr.2007.8.6.541 PMC2627457 18071285

[14] Chatzipantelis P Karvouni E Fragoulidis GP Voros D Pafiti A. Clinicopathologic features of two rare cases of mesenchymal metastatic tumors in the pancreas: review of the literature. Pancreas. (2006) 33:301–03. doi: 10.1097/01.mpa.0000234075.53630.2f 17003653

[15] Yamamoto H Watanabe K Nagata M Honda I Watanabe S Soda H. Surgical treatment for pancreatic metastasis from soft-tissue sarcoma: report of two cases. Am J Clin Oncol. (2001) 24:198–200. doi: 10.1097/00000421-200104000-00019 11319298

[16] Komatsu T Taira S Matsui O Takashima T Note M Fujita H. A case of ruptured mesenchymal chondrosarcoma of the pancreas. Radiat Med. (1999) 17:239–41. 10440114

[17] Byun GH Kang JH Kim JA Kim HK Lee KS Chang ED. Extraskeletal Mesenchymal Chondrosarcoma of Thigh with Metastasis to Pancreas: a case report and literature review. J Korean Cancer Assoc. (1995) 27:1070–77.

[18] Hu HJ Liao MY Xu LY. Primary retroperitoneal extraskeletal mesenchymal chondrosarcoma involving the vena cava: a case report. Oncol Lett. (2014) 7:1970–74. doi: 10.3892/ol.2014.2012 PMC4049765 24932271

[19] Paasch C De Santo G Boettge KR Strik MW. Mesenchymal chondrosarcoma metastasising to the pancreas. BMJ Case Rep. (2018) 11:e226369. doi: 10.1136/bcr-2018-226369 PMC6326294 30598468

[20] Cohen JN Solomon DA Horvai AE Kakar S. Pancreatic involvement by mesenchymal chondrosarcoma harboring the HEY1-NCOA2 gene fusion. Hum Pathol. (2016) 58:35–40. doi: 10.1016/j.humpath.2016.07.026 27544802

[21] Wang L Motoi T Khanin R Olshen A Mertens F Bridge J. Identification of a novel, recurrent HEY1-NCOA2 fusion in mesenchymal chondrosarcoma based on a genome-wide screen of exon-level expression data. Genes Chromosomes Cancer. (2012) 51:127–39. doi: 10.1002/gcc.20937 PMC3235801 22034177

[22] Hashimoto N Ueda T Joyama S Araki N Beppu Y Tatezaki S. Extraskeletal mesenchymal chondrosarcoma: an imaging review of ten new patients. Skeletal Radiol. (2005) 34:785–92. doi: 10.1007/s00256-005-0025-9 16211384

[23] Trembath DG Dash R Major NM Dodd LG. Cytopathology of mesenchymal chondrosarcomas: a report and comparison of four patients. Cancer. (2003) 99:211–16. doi: 10.1002/cncr.11300 12925982

[24] Taori K Patil P Attarde V Chandanshive S Rangankar V Rewatkar N. Primary retroperitoneal extraskeletal mesenchymal chondrosarcoma: a computed tomography diagnosis. Br J Radiol. (2007) 80:e268–70. doi: 10.1259/bjr/13711118 17989325

[25] Shapeero LG Vanel D Couanet D Contesso G Ackerman LV. Extraskeletal mesenchymal chondrosarcoma. Radiology. (1993) 186:819–26. doi: 10.1148/radiology.186.3.8430193 8430193

[26] Cesari M Bertoni F Bacchini P Mercuri M Palmerini E Ferrari S. Mesenchymal chondrosarcoma. An analysis of patients treated at a single institution. Tumori. (2007) 93:423–27. doi: 10.1177/030089160709300503 18038872

[27] Frezza AM Cesari M Baumhoer D Biau D Bielack S Campanacci DA. Mesenchymal chondrosarcoma: prognostic factors and outcome in 113 patients. A European Musculoskeletal Oncology Society study. Eur J Cancer. (2015) 51:374–81. doi: 10.1016/j.ejca.2014.11.007 25529371

[28] Dang HN Tran PA Dang TN Le TT Le VTT Nguyen H. Pancreatic metastasis of mesenchymal chondrosarcoma: a surgical case report and review of literature. Ann Med Surg (Lond). (2024) 86:580–87. doi: 10.1097/MS9.0000000000001549 PMC10783418 38222770

[29] Huvos AG Rosen G Dabska M Marcove RC. Mesenchymal chondrosarcoma. A clinicopathologic analysis of 35 patients with emphasis on treatment. Cancer. (1983) 51:1230–37. doi: 10.1002/(ISSN)1097-0142 6825046

[30] Louvet C de Gramont A Krulik M Jagueux M Hubert D Brissaud P. Extraskeletal mesenchymal chondrosarcoma: case report and review of the literature. J Clin Oncol. (1985) 3:858–63. doi: 10.1200/JCO.1985.3.6.858 2409241

[31] Johnson DB Breidahl W Newman JS Devaney K Yahanda A. Extraskeletal mesenchymal chondrosarcoma of the rectus sheath. Skeletal Radiol. (1997) 26:501–04. doi: 10.1007/s002560050274 9297757

[32] Kim GE Kim DK Park IJ Ro JY. Mesenchymal chondrosarcoma originating from the femoral vein. J Vasc Surg. (2003) 37:202–05. doi: 10.1067/mva.2003.106 12514602

[33] Fanburg-Smith JC Auerbach A Marwaha JS Wang Z Rushing EJ. Reappraisal of mesenchymal chondrosarcoma: novel morphologic observations of the hyaline cartilage and endochondral ossification and beta-catenin, Sox9, and osteocalcin immunostaining of 22 cases. Hum Pathol. (2010) 41:653–62. doi: 10.1016/j.humpath.2009.11.006 20138330

[34] Nakayama R Miura Y Ogino J Susa M Watanabe I Horiuchi K. Detection of HEY1-NCOA2 fusion by fluorescence *in-situ* hybridization in formalin-fixed paraffin-embedded tissues as a possible diagnostic tool for mesenchymal chondrosarcoma. Pathol Int. (2012) 62:823–26. doi: 10.1111/pin.12022 23252872

[35] Lee E Lee HY Choe G Kim KJ Lee WW Kim SE. Extraskeletal intraspinal mesenchymal chondrosarcoma; 18F-FDG PET/CT finding. Clin Nucl Med. (2014) 39:e64–66. doi: 10.1097/RLU.0b013e3182815cd5 23877507

[36] Bishop MW Somerville JM Bahrami A Kaste SC Interiano RB Wu J. Mesenchymal chondrosarcoma in children and young adults: a single institution retrospective review. Sarcoma. (2015) 2015:608279. doi: 10.1155/2015/608279 26146478 PMC4469840

