# Supplementary material for: Case report: Extraskeletal mesenchymal chondrosarcoma with a rare metastasis to the pancreas
Source: Front Oncol. 2024 Oct 9;14:1324732. doi: 10.3389/fonc.2024.1324732 (PMC11496084; doi:10.3389/fonc.2024.1324732)
Supplement: Supplementary Table 1 — Literature review of the published cases of primary/metastatic ESMC of the pancreas. (highlighted) added in this report. * Outcome: months or years of survival after surgery. ** Years of survival after the diagnosis of MC. △ HEY1:NCOA2 gene fusion was first identified in MC in 2012 (21). ★ Cases of the primary ESMC of the pancreas. CT, chemotherapy; RT, radiotherapy; NR, not recorded; NA, not applicable. [file SupplementaryFile1.pdf]

Additional Table 1. Literature review of published cases of the primary/metastatic ESMC of the pancreas.

NO	Reference/Year	Age(Y)/ gender	Size (cm)/ Location (head, body, tail)	Primary Site	Metastatic Site	**Pancreatic tumor with calcified deposit**	Treatment	Clinical outcome*	Latency Period for Pancreatic Metastasis (y)	*HEY1-NCOA2* gene fusion△
1	Byun et al. (17)/1995	36/Female	7.7×4.3×5/ Tail	Left thigh	Pancreas	NR	Distal pancreatectomy CT	NR	Synchronous	NA
2	Komatsu et al. (16)/1999	28/Female	2.5/ Tail	Meninges	Pancreas	Yes	Distal pancreatectomy	NR	17	NA
3	Yamamoto et al. (15)/2001	29/Male	NR/ Body, Tail	Left thigh	Pancreas, lung, testis, skin, chest wall	Yes	Distal pancreatectomy	Alive at 10 years	3	NA
4	Naumann et al. (2)/2002	24/Female	NR/ NR	Left lower abdomen	Pancreas, lung, rib, kidney, humerus, spine	NR	RT, CT	Alive at 7 years**	6	NA
5	Chatzipantelis et al. (14)/2006	26/Male	3.8×3.5/ Tail	Brain	Pancreas, lung, thigh	NR	Distal pancreatectomy	Alive at 9 years	9	NA
★6	Oh et al. (13)/2007	41/Male	13×12×7/ Body, Tail	Pancreas	—	Yes	Enucleation	NR	—	NA
★7	Bu et al. (12)/2010	34/Female	18×16/ Body, Tail	Pancreas	—	No	Surgical resection of pancreas body	Alive at 52 months	—	NA

							and tail			
8	Tsukamoto et al. (11)/2014	39/Male	5×6/ Body, Tail	Left buttock	Pancreas, lung, sacrum, ilium, ischium,	No	Distal pancreatectomy CT	Dead at 34 months	Synchronous	No gene test result
9	Guo et al. (1)/2015	40/Male	2×3×2/ Body	Femoral vein	Pancreas, lung, pleural, mediastinal and axillary node	Yes	Distal pancreatectomy	Alive at 9 years	3	No gene test result
10	Smith et al. (10)/2015	44/Female	NR/ Body	Chest wall	Pancreas	NR	Distal pancreatectomy CT	Alive at 24 months	21	No gene test result
11	Chen et al. (9)/2022	34/Male	3.4/ Tail	Chest wall	Pancreas	Yes	Distal pancreatectomy CT	Alive at 3 months	1	No gene test result
**12**	Hayashida S et al. (8)/2023	45/ Female	2.2/ Head, tail	obturator externus muscle	Pancreas	Yes	Pancreaticoduodenectomy Distal pancreatectomy	Alive at 28 months	Synchronous	Yes
**13**	**Present case**	**45/ Female**	**10×8/ Body, Tail**	**Left thigh**	**Pancreas, lung, groin**	**Yes**	**Distal pancreatectomy CT**	**Alive at 39 months**	**Synchronous**	**No gene test result**

The table originates from Chen et al (9) and Dang et al (28), with a new column (highlighted) added by this report.

* Outcome: months or years of survival after surgery. ** Years of survival after diagnosis of MC. △ *HEY1-NCOA2* gene fusion was first identified in MC in 2012 (21).

★ Cases of the primary ESMC of the pancreas. CT: chemotherapy; RT: radiotherapy; NR: not recorded. NA: not applicable.

Additional Table 2. Literature review of the number of published skeletal and extraskeletal MC and the duration of follow-up for ESMC metastasis (Pancreatic metastasis not included).

NO.	Reference/Year	MC		ESMC Metastasis	Mean follow-up (months)
		Bone origin	Extraskeletal origin		
1	1983/Huvos et al.(29)	30	5	16★	37.9 (1-528)
2	1985/Louvet et al.(30)	/	1	NO	6
3	1986/Nakashima et al. (5)	72	38	43△(78)	65 (22-209)
4	1993/Shapeero et al.(25)	2	5	2	(11-120)
5	1997/Johnson et al.(31)	/	1	NO	NR
6	2003/Trembath et al.(23)	1	3	2	(0-45)
7	2003/Kim et al.(32)	/	1	NO	3
8	2005/Hashimoto et al.(22)	/	10	2	54.5 (9-156)
9	2007/Taori et al.(24)	/	1	NO	/
10	2007/Cesari et al.(26)	13	8	18★	48 (7-237)
11	2010/Fanburg-Smith et al.(33)	15	7	1△(10)	(4-228)
12	2012/Shakked et al.(6)	18	2	1★	(0-336)
13	2012/Robert et al.(34)	6	4	3★	NA
14	2014/Lee et al.(35)	/	1	NO	NA
15	2015/Bishop et al.(36)	5	7	2★	78 (62-128)
16	2015/Frezza et al.(27)	72	41	45△(95)	179 (12-408)
17	2017/Schneiderman et al.(3)	82	123	NR	NR

Notes: △ The numbers in parentheses on the right indicate the total number of MC cases followed up, while the numbers on the left represent the cases in which metastasis occurred, including both skeletal and extraskeletal origins. ★ The number of cases in which metastasis occurred among all cases of MC, including both skeletal and extraskeletal origins. NR: not recorded. NA: not applicable.
